# A comparison of feature extraction methods for prediction of neuropsychological scores from functional connectivity data of stroke patients

**DOI:** 10.1186/s40708-021-00129-1

**Published:** 2021-04-20

**Authors:** Federico Calesella, Alberto Testolin, Michele De Filippo De Grazia, Marco Zorzi

**Affiliations:** 1 Department of General Psychology, University of Padova, 35131 Padova, Italy; 2 Department of Information Engineering, University of Padova, 35131 Padova, Italy; 3 IRCCS San Camillo Hospital, 30126 Venice-Lido, Italy

**Keywords:** Resting state networks, Functional connectivity, Machine learning, Feature extraction, Dimensionality reduction, Predictive modeling

## Abstract

Multivariate prediction of human behavior from resting state data is gaining increasing popularity in the neuroimaging community, with far-reaching translational implications in neurology and psychiatry. However, the high dimensionality of neuroimaging data increases the risk of overfitting, calling for the use of dimensionality reduction methods to build robust predictive models. In this work, we assess the ability of four well-known dimensionality reduction techniques to extract relevant features from resting state functional connectivity matrices of stroke patients, which are then used to build a predictive model of the associated deficits based on cross-validated regularized regression. In particular, we investigated the prediction ability over different neuropsychological scores referring to language, verbal memory, and spatial memory domains. Principal Component Analysis (PCA) and Independent Component Analysis (ICA) were the two best methods at extracting representative features, followed by Dictionary Learning (DL) and Non-Negative Matrix Factorization (NNMF). Consistent with these results, features extracted by PCA and ICA were found to be the best predictors of the neuropsychological scores across all the considered cognitive domains. For each feature extraction method, we also examined the impact of the regularization method, model complexity (in terms of number of features that entered in the model) and quality of the maps that display predictive edges in the resting state networks. We conclude that PCA-based models, especially when combined with L1 (LASSO) regularization, provide optimal balance between prediction accuracy, model complexity, and interpretability.

**Supplementary Information:**

The online version contains supplementary material available at 10.1186/s40708-021-00129-1.

## Introduction

Resting State Functional Connectivity (RSFC) represents the correlation in the spontaneous fluctuations of the blood oxygen level-dependent signal between brain regions, measured at rest using functional magnetic resonance imaging (fMRI) [1–3]. One important goal of current neuroimaging research is to associate individual RSFC with behavior. Predictive modeling of individual differences from neuroimaging data is particularly attractive in the context of neurological or psychiatric disorders, with potential applications to prediction of long-term behavioral outcomes or response to intervention [4]. In stroke patients, RSFC has been successfully employed to predict individual deficits in several cognitive domains, such as language, visuo-spatial memory, verbal memory, and attention [5, 6].

Machine learning has been a key enabling technology for investigating brain–behavior associations, because the analysis of neuroimaging data requires the adoption of multivariate approaches that can efficiently operate over high-dimensional feature spaces [7–9]. At the same time, neuroimaging datasets typically have a much greater number of features than observations [8, 10], which raises the risk of overfitting, that is, extracting rules or statistical patterns that specifically describe the training data but cannot be generalized to new observations [11, 12]. One possible way to mitigate the overfitting issue is to adopt regularization methods. For example, regularized regression methods such as ridge regression [6], elastic-net [13], and least absolute shrinkage and selection operator (LASSO) [14] include a penalty term that pushes the estimated coefficients of irrelevant features toward zero [15]. Besides limiting multicollinearity and overfitting, this often also improves model interpretability [13, 16, 17], making regularized algorithms particularly suitable for the analysis of neuroimaging data (for a recent review, see [18]). Another useful approach to tackle the “curse of dimensionality” in neuroimaging data is to first apply unsupervised dimensionality reduction techniques [8, 10, 19], to extract a limited number of features that can compactly describe the data distribution.

However, both regularized regression methods and feature extraction techniques can vary in performance, depending on the type of data and the task [10, 18], calling for a systematic assessment of the differences between these methods on neuroimaging data. Some recent works have compared the performance of several machine learning algorithms [18], and their interaction with dimensionality reduction methods [20]. Nonetheless, to the best of our knowledge, a similar approach has not yet been applied to multiple unsupervised feature extraction techniques.

**Fig. 1:**
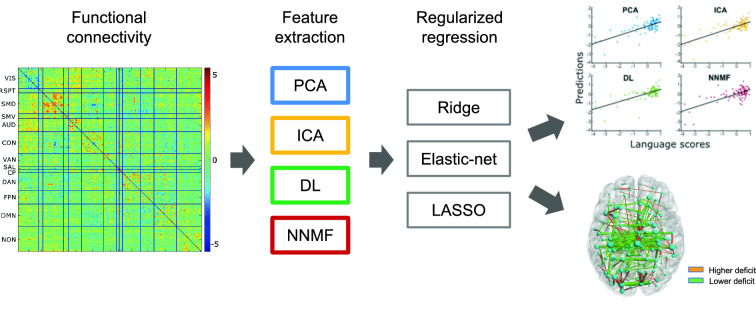
Machine learning pipeline for the prediction of neuropsychological scores from Resting State Functional Connectivity (RSFC) matrices of stroke patients. The mean RSFC matrix ($324 \times 324$) across all patients is shown as reference. Parcels in the matrix are sorted in relation to 12 large-scale intrinsic brain networks. Model predictions can be validated against left-out empirical data (top-right panels), while the most predictive edges can be visualized in a brain-like topology to better understand the contribution of different circuits to the behavioral deficits (bottom-right panel)

The goal of the present work is to systematically explore the impact of regularization in combination with different dimensionality reduction techniques, to establish which method can be more effective to build predictive models of behavioral outcome from RSFC. In particular, we used RSFC data from a relatively large and heterogeneous cohort of stroke patients [21] to predict the neuropsychological scores using a machine learning framework. In a first step, the RSFC matrices underwent a feature extraction analysis, implemented through different unsupervised dimensionality reduction methods: Principal Component Analysis, Independent Component Analysis, Dictionary Learning and Non-Negative Matrix Factorization. In a second step, the extracted features were entered as predictors into a regularized regression model . We used the elastic-net, a regularized regression method that linearly combines the L1 and L2 penalties of the LASSO and ridge methods, thereby allowing maximum flexibility in the choice of regularizer. Nevertheless, we also examined models restricted to “pure” L1 (LASSO) or L2 (ridge) regularization to assess the impact of the regression method as well as the potential interaction with the feature extraction methods (see Fig. 1 for a graphical illustration of the analysis pipeline). Finally, we compared the classic leave-one-out cross-validation with the more complex “nested” cross-validation scheme for models’ hyper-parameter tuning [22], which potentially leads to a more conservative estimate of model performance. Note that previous work on the same stroke dataset has only used Principal Component Analysis combined with ridge regression and non-nested cross-validation [5, 6].

The results section is organized as follows. First, we report results in the prediction of language scores. Language deficits are a very frequent outcome of stroke and their neural correlates show lower inter-individual variability in comparison to other cognitive functions like memory [6], thereby offering an ideal platform for systematic comparison of the different approaches (also see [23]). Prediction of language deficits in stroke has also been a main focus of studies that applied machine learning on structural lesion images [24–26]. In addition to reporting predictive accuracy, for each feature extraction method, we examined model complexity (in terms of the final number of features that entered in the model) and quality of the predictive maps obtained by back-projecting the regression weight to display the most predictive RSFC edges. We then extend our assessment on two additional neuropsychological scores that index verbal memory and spatial memory. Note that memory has a more distributed neural basis and the prediction of deficits from structural lesions is relatively poor compared to other behavioral domains [5]. Therefore, prediction of memory scores represents an important benchmark for RSFC-based machine learning methods.

## Materials and methods

### Participants and data acquisition

RSFC data were taken from a previously published study [6], which is the largest RSFC dataset available for stroke patients. The study included 132 symptomatic stroke patients who underwent a 30-minute-long RS-fMRI acquisition, 1–2 weeks after the stroke occurred. 32 subjects were excluded either for hemodynamic lags or excessive head motion. Functional connectivity can be represented with a symmetric matrix that captures the correlation structure between individual brain regions, defined according to a specific parcellation. In our case, for each patient, a RSFC matrix (of size $324 \times 324$) was calculated across 324 cortical parcels [27] (Fig. 1). The matrices were then vectorized, resulting in 52,326 FC values per subject. After fMRI acquisition, all participants underwent a behavioral assessment spanning several cognitive domains.

In this work, we focus on three different cognitive domains: language, spatial memory and verbal memory. Neuropsychological scores for these domains are available for different subsets of the participants. For the language domain ($n=95$), we used an overall “language factor” score [6] which captures the shared variance of several sub-tests (first principal component accounting for 77.3% of variance). In the memory domain, the first two components accounted for 66.2% of variance and were associated with spatial ($n=78$) and verbal ($n=78$) memory, respectively. All scores were normalized to represent impaired performance with negative values.

### Unsupervised feature extraction

Since the feature extraction process was unsupervised, in this phase, the entire dataset was used (here $n=100$ and $p=52,326$), regardless of the availability of the neuropsychological score. All the employed feature extraction methods aim to find a weight matrix *W* that can linearly transform the original $n \times p$ data matrix *X* in a new set of *k* features, with $k<p$ and usually $k<n$ , such that

1 $$\begin{aligned} F=XW, \end{aligned}$$

where *F* is the new feature space, and the parameter *k* is the number of features to be extracted. Since choosing the value of *k* is non-trivial, we systematically varied *k* from 10 to 95, with step size = 5, which resulted in 18 feature sets for each employed technique. The original data can be reconstructed by back-projecting the new feature set in the original space:

2 $$\begin{aligned} X_R=FW^T, \end{aligned}$$

where $X_R$ is the reconstructed data. To compare the compression ability of the feature extraction methods, the reconstruction error was calculated as the mean squared error (MSE) between *X* and $X_R$, for each value of *k*.

#### Principal component analysis (PCA)

PCA linearly transforms the original data into a smaller set of uncorrelated features called principal components, sorted by the data variance they explain [28]. First, *X* must be centered [29], so that it has zero-mean. PCA then searches for the eigenvalues and eigenvectors of the $p \times p$ covariance matrix $X^{T}X$. Hence, matrix factorization via singular value decomposition is applied, such that

3 $$\begin{aligned} X=UDW^T, \end{aligned}$$

where *U* is an $n \times n$ matrix containing the eigenvectors of $\text{XX}^T$, *D* is an $n \times p$ matrix with the square root of the eigenvalues on the diagonal, and *W* is a $p \times p$ matrix containing the eigenvectors of $X^{T}X$. However, if $p>n$, there are only $n-1$ non-zero eigenvalues, so only the first $n-1$ columns of *D* and *W* are kept [29]. Eigenvectors are sorted in descending order of explained variance. Hence, *W* contains $n-1$ principal components, expressed as a set of *p* weights that can map the original variables in a new compressed space. Since PCA is the only deterministic method we explored, it was performed only once and the first *k* features were then iteratively selected. For the other methods, the procedure had to be run repeatedly for each value of *k*. The *pca* MATLAB function was used.

#### Independent component analysis (ICA)

ICA assumes that a *p*-dimensional signal vector $X_{i,*}^{T}$ is generated by a linear combination of *k* sources (with $k \le p$), contained in vector $F_{i,*}^{T}$. The sources are assumed to be latent, independent and non-Gaussian [30]. Therefore,

4 $$\begin{aligned} X_{i,*}^{T}=AF_{i,*}^{T}, \end{aligned}$$

where *A* is a $p \times k$ unmixing matrix, which maps the signal in the sources. Hence, the sources are obtained by

5 $$\begin{aligned} F_{i,*}^{T}=WX_{i,*}^{T}, \end{aligned}$$

where *W* is the inverse of the unmixing matrix *A*. Then $F_{i,*}^{T}$ represents *k* latent independent features [30, 31]. To simplify the ICA problem, the data distribution is first centered, and then pre-processed through whitening so that a new vector $X_{i,*}^{T}$ with uncorrelated components and unit variance is obtained. In this case, PCA was used for data whitening [31]. The *FastICA* function of the scikit-learn library was used.

#### Dictionary learning (DL)

The DL algorithm, sometimes known as sparse coding, jointly solves for a $p \times k$ dictionary *W* and the new set of features *F* that best represent the data. However, an $L_1$ penalty term is included in the cost function, to obtain only few non-zero entrances. Hence, the cost function becomes

6 $$\begin{aligned}&(W,F)=\min _{(W,F)}\frac{1}{2}\Vert {X-FW^T}\Vert _{2}^{2}+\lambda \Vert {F}\Vert _1,\\& \text {subject to} \ \Vert {W_j}\Vert _2\le 1, \ \forall \ j=1,\ldots , k, \end{aligned}$$

where $\lambda$ is the $L_1$ penalty coefficient, controlling for the sparsity of the compressed representation [32]. The *Dictionary Learning* function of the scikit-learn library was used.

#### Non-negative matrix factorization (NNMF)

NNMF is a form of matrix factorization into non-negative factors *W* and *H* [33, 34], such that the linear combination of each column of *W* weighted by the columns of *H* can approximate the original data *X*:

7 $$\begin{aligned} X \approx WH. \end{aligned}$$

To do that, the NNMF aims to minimize the following loss function:

8 $$\begin{aligned} \begin{aligned} \Vert {X-WH}\Vert _{F}^{2}, \ \text{subject to} \ W,H \ge 0. \end{aligned} \end{aligned}$$

The *nnmf* MATLAB function with the “multiplicative update algorithm” was used.

### Regularized regression

The feature sets extracted by each method were then used as regressors for the prediction of the neuropsychological scores. Note that only the subjects with available score were kept in this phase (see sect. 2.1 above). The regressors were first standardized, and then entered into the elastic-net penalized regression [13, 17, 35] (the MATLAB *lasso* function was used). The elastic-net regression solves for

9 $$\begin{aligned} \min _{\beta } \left( \frac{1}{2n}\sum _{i=1}^{n}(y_i-x_{i}^{T}\beta )^2 + \lambda P_{\alpha }(\beta )\right) , \end{aligned}$$

where *n* is the number of observations, $y_i$ is the prediction target at observation *i*, $x_i$ is the data observation *i* with *p* variables, $\lambda$ is the non-negative regularization coefficient, $\beta$ is the *p* regression coefficient and $P_\alpha$ is defined as

10 $$\begin{aligned} P_{\alpha }(\beta )=\sum _{j=1}^{p}\left( \frac{1}{2}(1-\alpha )\beta _{j}^{2}+\alpha \vert {\beta _j}\vert \right) . \end{aligned}$$

Therefore, the elastic-net loss function requires two free parameters to be set, namely the $\lambda$ and $\alpha$ parameters. The $\lambda$ parameter regulates the penalization strength, so the larger the $\lambda$, the more coefficients are shrunk toward zero. The $\alpha$ parameter sets the regularization type: with $\alpha =1$, an $L_1$ penalization (LASSO) is obtained, whereas with $\alpha \approx 0$, the $L_2$ penalty (ridge regression) is approached [36]. The main difference is that LASSO forces the coefficient estimates to have exactly zero values, whereas the ridge regularization shrinks the coefficients to near-zero values [16]. Lastly, the elastic-net regression combines both the penalization terms [36]. The $\lambda$ was tuned over 100 possible values, logarithmically spaced between $10^{-5}$ and $10^5$. The considered set of $\alpha$ values was 0.001, 0.25, 0.5, 0.75, and 1.

### Cross-validation setup and model estimation

To find optimal hyper-parameters, it is common practice to employ a grid-search procedure with cross-validation (CV). We tested the combinations of possible values for all hyper-parameters (*k*, $\lambda$ and $\alpha$) using a Leave-One-Out (LOO) scheme: the grid search was repeated for *n* iterations, where *n* is the number of subjects. At each CV iteration, a different subject was removed from the sample, and the remaining *n-1* subjects (training set) were used to estimate the coefficients with each parameter combination. Each model was then used for the prediction of the neuropsychological score of the left-out subject (test set), and the difference between the prediction and the true value was recorded. The combination of hyper-parameters leading to the model with lowest MSE was selected as the “best model”. Note that a constraint was implemented on the parameter *k*, to avoid to select models with $k>n$.

In the standard LOO, however, selection of the best model is based only on the test error, which could lead to optimistically biased model performance [8]. To compare the standard LOO procedure with a more sophisticated (but computationally more expensive) cross-validation scheme, for the case of the language score, we also implemented a *nested* LOO (n-LOO) CV. In this case, the hyper-parameters are tuned on different observations from that of the test set: the *n–1* training set is iteratively further decomposed into a *n–2* training set and a left-out subject, called validation set. As a consequence, selection of the best model is based on the minimization of the error calculated on the validation set. Once the best model is selected within the inner loop, it is applied to the test set to measure the final performance [8, 19, 35]. A drawback of this approach is that it can lead to the choice of different models across the CV loops: to produce the final model of the n-LOO procedure, three measures of central tendency were used for choosing the optimal hyper-parameters, namely mean (n-average condition), median (n-median condition) and mode (n-mode condition).

### Performance measures and model comparison

To assess model performance and compare the models generated by the different feature extraction methods, we report both $R^{2}$ and MSE. The $R^{2}$ was computed as

11 $$\begin{aligned} R^2=1-\frac{\sum (Y - Y')^2}{\sum (Y-\overline{Y})^2}, \end{aligned}$$

where *Y* are the observed behaviour scores, $Y'$ are the predicted behavioural scores, and $\overline{Y}$ is the mean of the observed behavioural scores. Moreover, we computed the Bayesian information criterion (BIC) [37] to provide a measure of fit that takes model complexity into account (note that only the non-zero coefficients were used for BIC calculation). Potential differences in the distributions of the quadratic residuals were statistically tested through the Wilcoxon signed rank test [38], corrected for multiple comparisons using the Bonferroni method. Finally, for each method, the optimal regression coefficients were back-projected in the original space, by means of linear transformation through the features’ weights, and restored in a symmetric matrix. This provides a map that displays the predictive edges in the resting state networks. To visualize critical connectivity patterns related to each cognitive impairment we also represented the most important edges (top 200 in absolute value) using a brain-like topology (see rightmost part of Fig. 1).

The complete source code used to perform the analyses presented in this article is made freely available online (see section “Availability of data and materials”).

## Results

**Fig. 2:**
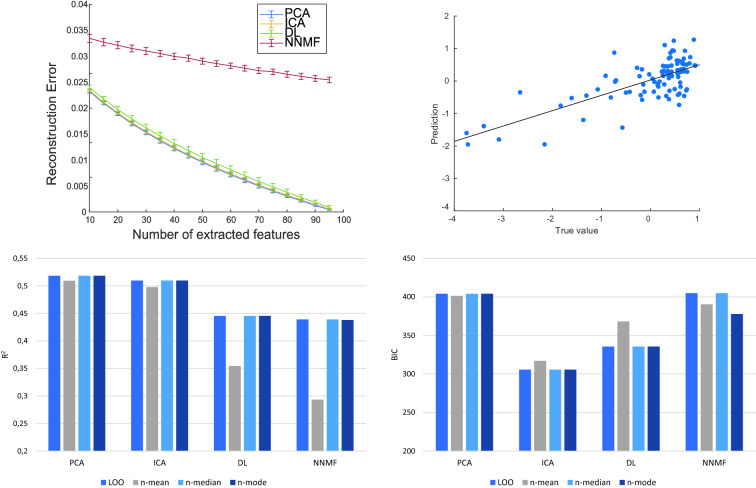
Top left: reconstruction error for each dimensionality reduction method as a function of the number of extracted features. Top right: PCA-based model predictions of language scores with LOO CV. Bottom: ${R}^{2}$ and BIC differences across the CV schemes for each feature extraction method

The feature extraction methods were first assessed based on their reconstruction error. For all methods, the reconstruction error decreased when increasing the number of features (Fig. 2, top-left panel). PCA and ICA showed the lowest reconstruction error, suggesting a higher compression ability of these methods. DL performed slightly worse, and NNMF showed generally higher reconstruction error.

In the language domain, PCA and ICA features yielded the best prediction accuracy, whereas DL- and NNMF-based models explained 6–7% less variance (Table 1; also see Fig. 2 top-right panel for a graphical illustration of the PCA-based model predictions). Despite PCA and ICA having very similar $R^{2}$ values, the ICA-based model showed better performance when considering the BIC value because of its smaller number of parameters (i.e., features entering in the final model). However, no significant difference between the squared residuals of the models was detected by the Wilcoxon signed rank test (all $p\; > \;0.05/6$).

We also examined the effect of the CV scheme upon model performance (Fig. 2, bottom). In the nested CV scheme, by averaging the hyper-parameters (n-mean condition), PCA ($R^{2} = 0.51$; $\text {MSE}=0.49$) and ICA ($R^{2} = 0.50; \text {MSE}=0.50$) showed only a marginal decrease of the performance, whereas a larger contraction of the predictive accuracy was observed in the DL- ($R^{2} = 0.35; \text {MSE}=0.64$) and NNMF- ($R^{2} = 0.29; \text {MSE}=0.70$) based models. In the n-mode condition, the final models yielded the same performance as those selected in the LOO setup, except for the NNMF-based model. However, the resulting performance ($R^{2} = 0.44; \text {MSE}=0.56$) decreased only by 0.09% compared to the LOO scheme. Finally, the n-median condition was the most consistent across methods and yielded the same level of performance obtained in the standard LOO setup. It appears, therefore, that the measure of central tendency used for choosing the final model in the n-LOO scheme can affect the performance. The predictive model can be poor when averaging the parameters across subjects, whereas choosing the median (or mode) allows to achieve the same performance level obtained using the simpler LOO scheme. This finding can be explained by the high susceptibility of the mean to outliers, so that major departures from the distribution of the selected parameters could drive the mean toward the outlier values. In this case, the median represents a more stable measure of central tendency. In light of the comparable performance yielded by LOO and n-LOO (especially for the n-median condition), we only considered the simpler and computationally lighter LOO scheme for extending our investigation to the prediction of verbal and spatial memory scores.

**Table 1:** Results of elastic-net regression

Cognitive domain	Method	$R^{2}$	MSE	BIC	$\lambda$	$\alpha$	*k*	NZ
Language ($n=95$)	PCA	0.52	0.48	404.10	0.22	0.001	45	45
	ICA	0.51	0.49	305.60	0.11	0.25	25	23
	DL	0.45	0.55	335.52	0.09	0.25	30	27
	NNMF	0.44	0.56	404.95	0.04	0.50	45	42
Spatial memory ($n=78$)	PCA	0.23	0.76	295.45	0.11	1	50	22
	ICA	0.24	0.75	395.18	0.56	0.001	45	45
	DL	0.20	0.79	285.88	0.09	1	40	19
	NNMF	0.21	0.78	371.87	0.09	0.75	75	39
Verbal memory ($n=78$)	PCA	0.34	0.65	327.62	0.09	0.75	45	32
	ICA	0.28	0.72	391.29	0.44	0.001	45	45
	DL	0.18	0.81	444.48	0.56	0.001	55	55
	NNMF	0.10	0.88	451.38	1.42	0.001	55	55

Performance of elastic-net regression models in the prediction of neuropsychological scores as a function of the feature extraction method. The value of the optimized parameters ($\lambda$, $\alpha$, and *k*) and the number of non-zero features (NZ) are also reported. $R^{2}$: percentage of variance explained.

* MSE* mean squared error,* BIC* Bayesian information criterion

**Fig. 3:**
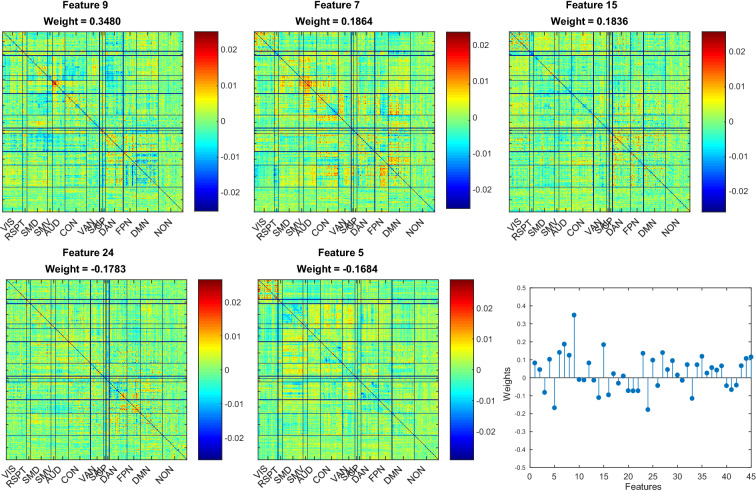
The 5 features associated with the highest regression coefficients (absolute value) in the PCA-based model for the prediction of the language scores, and model regression coefficients

**Fig. 4:**
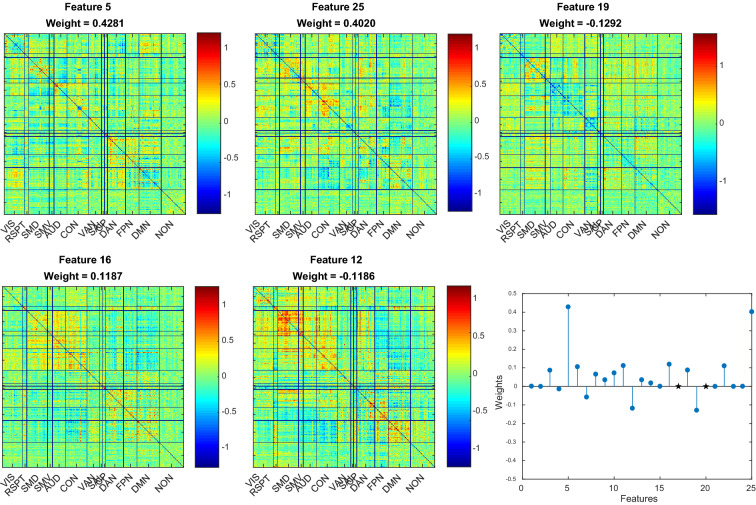
The 5 features associated with the highest regression coefficients (absolute value) in the ICA-based model for the prediction of the language scores, and model regression coefficients. Black stars represent $\text {coefficients}=0$

**Fig. 5:**
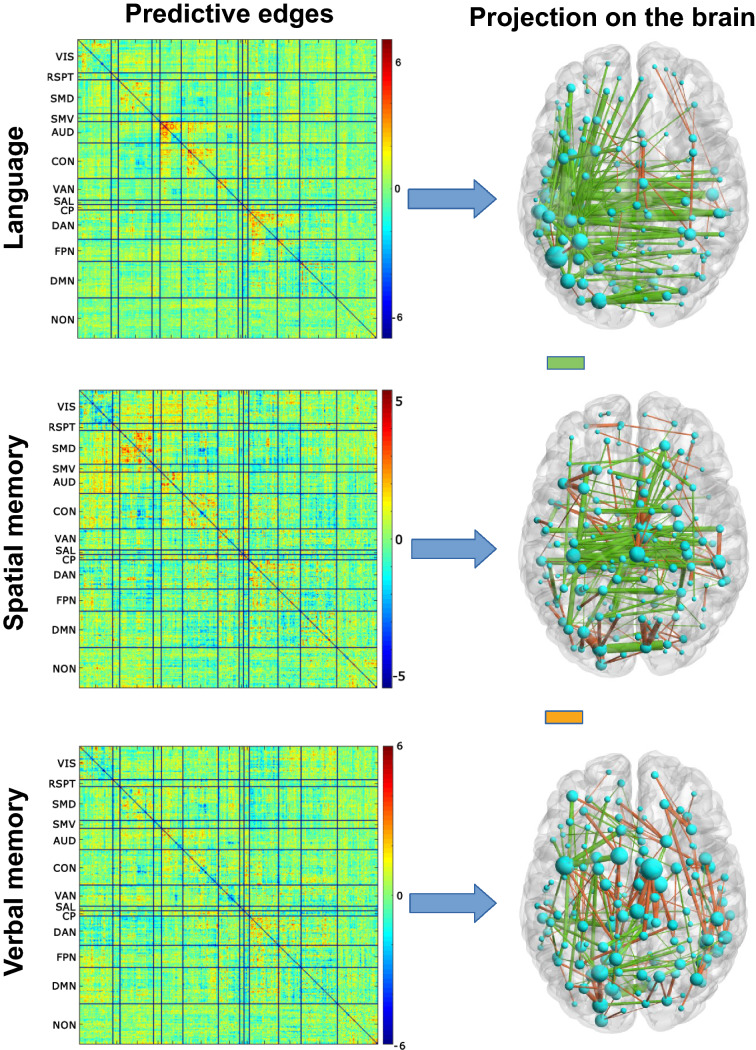
Maps of predictive functional connectivity edges for PCA-based models obtained by back-projecting the regression coefficients. The top 200 edges are projected onto a semitransparent brain: green connections represent positive weights, whereas orange connections represent negative weights. The spheres/nodes represent the cortical parcels linked by the selected edges and are displayed with size proportional to their contribution to the model

For each method, we then examined the model regression coefficients to highlight the features associated with the strongest weights, which in turn drive the model predictions (Fig. 3 for PCA; Fig. 4 for ICA; Additional file 1: Fig. S1 for DL; Additional file 2: Fig. S2 for NNMF). Comparison of the top features in the PCA- and ICA-based models reveals good consistency across methods and highlights the importance of functional connectivity in the auditory network for the prediction of language scores (also see Additional file 1: Fig. S1 for DL and Additional file 2: Fig. S2 for NNMF). Moreover, for each method, we back-projected the model regression coefficients into the original space to assess the quality of the predictive maps (Fig. 5, top panel; see Additional file 3: Fig. S3 for ICA, DL and NNMF): the resulting structures look fairly similar, and the matrices are indeed highly correlated (${r}_{\text{PCA-ICA}} = 0.84$; ${r}_{\text{PCA-DL}} = 0.72$; ${r}_{\text{ICA-DL}} = 0.71$), with the exception of the NNMF-based model (${r}_{\text{NNMF-PCA}} = 0.58$; ${r}_{\text{NNMF-ICA}} = 0.58$; ${r}_{\text{NNMF-DL}} = 0.44$). In particular, connectivity patterns in the auditory, cingulo-opercular, dorsal attentional and fronto-parietal networks seem to be particularly relevant for the prediction of language scores.

When predicting the spatial memory score, an analogous pattern to that of the language domain emerged. PCA and ICA features reached the best performance with similar ${R}^{2}$ values, followed by DL and NNMF (Table 1). Nonetheless, the regression based on PCA allowed to select fewer parameters than ICA, resulting in a lower BIC value. Also in this case, the Wilcoxon signed rank test did not show any significant difference between the models (all $p\;>\;0.05/6$). Furthermore, the back-projected coefficients (Fig. 5, middle panel; see Additional file 3: Fig. S3 for ICA, DL and NNMF back-projected coefficients) were highly correlated between the PCA and ICA models (${r}_{\text{PCA-ICA}} = 0.77$) and between the ICA and DL models (${r}_{\text{ICA-DL}} = 0.71$). The correlation values were instead smaller between PCA and DL (${r}_{\text{PCA-DL}} = 0.59$), and NNMF correlated poorly with all other methods (${r}_{\text{NNMF-PCA}} = 0.20$; ${r}_{\text{NNMF-ICA}} = 0.17$; ${r}_{\text{NNMF-DL}} = 0.39$). Notably, some relevant intra-network connectivity pattern associated with the performance in the spatial memory domain can be identified, such as dorsal and ventral somato-motor networks, cingulo-opercular network, and auditory network. The PCA features associated to the strongest regression weights are shown in Additional file 4: Fig. S4.

The features extracted by PCA were the best predictors also for the prediction of the verbal memory score. ICA yielded a slightly worse performance (explaining 6% less variance), and the PCA-based model also retained fewer parameters leading to a lower BIC value (Table 1). In the DL- and NNMF-based models, the ${R}^{2}$ dropped by 16% and 23%, respectively. Despite the differences in the predictive accuracy, no significant differences was found across the models (all ${p}>0.05/6$). Back-projection of the coefficients (Fig. 5, bottom panel; see Additional file 3: Fig. S3 for ICA, DL and NNMF results) produced maps that were highly correlated across the PCA, ICA and DL methods (${r}_{\text{PCA-ICA}} = 0.86$; ${r}_{\text{PCA-DL}} = 0.80$; ${r}_{\text{ICA-DL}} = 0.90$), whereas the NNMF-based model did not show notable correlations (${r}_{\text{NNMF-PCA}} = 0.46$; ${r}_{\text{NNMF-ICA}} = 0.56$; ${r}_{\text{NNMF-DL}} = 0.57$). Intra-network connectivity in the dorsal somato-motor, auditory, cingulo-opercular, and ventral and dorsal attentional networks appears to be particularly relevant for the prediction of the neuropsychological score in the verbal memory domain. The PCA features associated with the strongest regression weights are shown in Additional file 5: Fig. S5.

We finally assessed the predictive accuracy obtained with the different feature extraction methods when the regularized regression method was kept constant by forcing the $\alpha$ parameter to be either 0.001 (yielding ridge regression) or 1.0 (yielding LASSO regression) (Table 2). The results are aligned with those in which $\alpha$ was optimized. Nevertheless, the type of regularization appears to interact with the feature extraction method. For instance, in the language domain, the PCA-based model achieved marginally superior ${R}^{2}$ value with $\alpha =0.001$ but for verbal memory the $\alpha =1$ model was markedly superior. For the spatial memory score, the predictive accuracy was equivalent between the two values of $\alpha$. ICA reached the best performance with $\alpha =0.001$ both in the spatial and verbal memory domains. In the language domain instead, the ${R}^{2}$ values were very similar. The predictive accuracy of DL appeared to be independent of the value of $\alpha$ when predicting the language and verbal memory scores. However, in the verbal memory domain, the ${R}^{2}$ dropped when $\alpha =1$. The predictive accuracy of NNMF was similar between the two $\alpha$ values both in the language and verbal memory domains, whereas a slightly greater gap emerged in the prediction of the spatial memory score, suggesting that the LASSO solution was more suitable. Overall, PCA was the best performing method across cognitive domains and for the two memory scores this was obtained using LASSO regularization (with identical performance to the more flexible elastic-net models). For the language domain, the advantage of the $\alpha =0.001$ model over the LASSO model in terms of ${R}^{2}$ was marginal (3%) and it was offset by the larger number of parameters, as also indexed by the lower BIC value of the latter model.

**Table 2:** Results with $\alpha =0.001$ and $\alpha =1$

Cognitive domain	Method	$\alpha =0.001$				$\alpha =1$			
		${R}^{2}$	MSE	BIC	NZ	${R}^{2}$	MSE	BIC	NZ
Language ($n=95$)	PCA	0.52	0.48	404	45	0.49	0.51	365	35
	ICA	0.49	0.50	364	35	0.50	0.49	303	22
	DL	0.43	0.57	352	30	0.43	0.57	366	33
	NNMF	0.42	0.57	422	45	0.44	0.56	378	36
Spatial memory ($n=78$)	PCA	0.23	0.76	396	45	0.23	0.76	295	22
	ICA	0.24	0.75	395	45	0.19	0.80	344	32
	DL	0.20	0.79	421	50	0.20	0.79	286	19
	NNMF	0.15	0.84	403	45	0.20	0.79	364	37
Verbal memory ($n=78$)	PCA	0.27	0.72	501	70	0.34	0.65	319	30
	ICA	0.28	0.72	391	45	0.19	0.80	378	40
	DL	0.18	0.81	444	55	0.07	0.91	297	19
	NNMF	0.10	0.88	451	55	0.08	0.91	266	12

Performance of regularized regression with the $\alpha$ parameter fixed at 0.001 (ridge) and 1 (LASSO) in the prediction of neuropsychological scores as a function of the feature extraction method. The number of non-zero features (NZ) is also reported. ${R}^{2}$: percentage of variance explained

* MSE* mean squared error,* BIC* Bayesian information criterion

## Discussion

In this work, we systematically compared four unsupervised dimensionality reduction methods in their ability to extract relevant features from RSFC matrices. In particular, we assessed how different methods influenced a regularized regression model trained on the RSFC features to predict the cognitive performance of stroke patients.

Overall, PCA and ICA appeared to be the best methods for extracting robust predictors, which is consistent with the greater compression ability exhibited by these methods, compared to DL and NNMF. A greater compression capacity is indeed related to a better representation of the data, and so to a higher amount of information retained in the encoding space.

Though PCA- and ICA-based models had similar performance, PCA might be overall preferable. Indeed, the PCA-based model reached the best performance in the prediction of both the language and verbal memory scores, and it also approached the predictive accuracy of the ICA-based model when predicting the spatial memory score. Furthermore, in the spatial and verbal memory domains, the PCA-based model relied on fewer parameters than ICA. This facet should not be underestimated since a reduced number of descriptors improves model interpretability and might also allow to better generalize to out-of-sample predictions. In contrast, ICA relied on fewer features for the prediction of the language scores. However, considering the PCA-based models in the language domain, the variation of the ${R}^{2}$ between the ridge-approaching and LASSO solutions was quite narrow and the latter model was markedly more parsimonious. Moreover, LASSO regression on PCA features yielded the same performance level of the more flexible elastic-net regression for both verbal and spatial memory domains. This suggests that many PCA features can be discarded without losing large amounts of predictive accuracy. It is also noteworthy that ICA instead showed a more significant decrease in ${R}^{2}$ in the spatial and verbal memory, when forcing a LASSO solution.

Despite the differences across the feature extraction methods, we did not observe any significant difference between the final predictive models when compared in terms of residuals. Furthermore, we observed high correlations between the back-projected predictive maps, except for NNMF, which was less aligned with the other methods. This is probably due to the non-negativity constraint applied on the transformation matrix. Overall, these results suggest that PCA, ICA and DL extract similar structure from the RSFC matrices. Inspection of the predictive maps suggested that the language score was associated with functional connectivity in the auditory, cingulo-opercular, dorsal attentional and fronto-parietal networks. The prediction of the neuropsychological score in the spatial memory domain was associated with the dorsal and ventral somato-motor networks, the auditory network and the cingulo-opercular network. Finally, the dorsal somato-motor network, auditory network, cingulo-opercular network, and ventral and dorsal attentional networks appeared to be relevant for the prediction of the verbal memory score.

Previous studies that used machine learning to predict the cognitive performance of stroke patients applied PCA on the RSFC matrices and retained all principal components that cumulatively explained 95% of the variance as features for (non-nested) cross-validated ridge-penalized regression [5, 6]. Here we did not set any *a priori* constraints on the number (and type) of features as well as on the type of regularization, opting instead for a more data-driven approach. It is, therefore, valuable to compare results across studies based the same dataset. Notably, the present PCA-based models systematically outperformed the predictive accuracy of the models reported in the recent work of Salvalaggio et al. [5]. Moreover, the number of PCA features retained in the previous work was much higher (range 64–79) compared to the present PCA models (range 22–45 for the same cognitive domains). The number of features was less than half (range 22–35) for the PCA + LASSO solution. Overall, this suggests that PCA combined with L1-regularized (LASSO) regression provides optimal balance between predictive accuracy and model complexity. A further advantage of PCA over ICA is the lower computational burden, also because PCA is computed independently of the number of components that are later selected for regression.

The analyses carried out on the language score also compared a standard cross-validation scheme with a nested cross-validation approach. The latter is usually considered as more appropriate because it prevents the potential performance inflation induced by tuning the model hyperparameters on the test set: nested cross-validation should lead to a more conservative estimate of the generalization performance of the predictive model [39]. However, in the language domain, we did not find any difference in performance between the nested and non-nested cross-validation approaches when using median or mode as criteria for choosing optimal hyper-parameters. This suggests that the non-nested setup could still lead to the selection of optimal models that can generalize to new observations, but with a much less intensive computational burden (see also [40] for an extensive empirical assessment of the performance difference between nested and non-nested CV approaches).

Future studies should further extend our results to other data and tasks. For instance, the impact of the feature extraction method might also be evaluated for other types of neuroimaging data available for stroke patients, such as EEG connectivity measures [41] or 3D images of brain lesions [23]. Moreover, despite our approach allows building robust models even with limited samples, further efforts should be spent in creating larger-scale datasets, which would allow to deploy even more powerful predictive models, such as those based on deep learning [42].

## Conclusion

Type of data and task are known to potentially affect the performance of both regularized regression and feature extraction techniques. In this work, we compared the ability of four unsupervised dimensionality reduction methods to extract meaningful features from RSFC data of stroke patients. The goodness of the extracted features was assessed based on their capacity to predict the neuropsychological scores of the patients in three cognitive domains (i.e., language, spatial memory, and verbal memory) by means of different regularized regression methods. Our results suggest that a machine learning pipeline based on PCA and regularized regression method promoting feature selection is the preferable method. Besides yielding the highest predictive accuracy, its sparse solution promotes model simplicity and interpretability. Overall, our methodological approach allows to draw solid conclusions in relation to the optimal machine learning pipeline that should be used to build predictive models of neuropsychological deficits to strike a balance between accuracy and model complexity, which is of crucial importance given the strong translational implications of this kind of tools.

## Supplementary Information

**Additional file 1: Figure S1.** The 5 features associated to the highest regression coefficients (absolute value) in the DL-based model for the prediction of the language scores, and model regression coefficients. Black stars represent coefficients = 0.

**Additional file 2: Figure S2.** The 5 features associated to the highest regression coefficients (absolute value) in the NNMF-based model for the prediction of the language scores, and model regression coefficients. Black stars represent coefficients = 0.

**Additional file 3: Figure S3.** Maps of predictive functional connectivity edges for ICA-, DL- and NNMF-based models obtained by back-projecting the regression coefficients. DL: Dictionary Learning; ICA: Independent Component Analysis; NNMF: Non-Negative Matrix Factorization.

**Additional file 4: Figure S4.** The 5 features associated to the highest regression coefficients (absolute value) in the PCA-based model for the prediction of the neuropsychological scores in the spatial memory domain, and model regression coefficients. Black stars represent coefficients = 0.

**Additional file 5: Figure S5.** The 5 features associated to the highest regression coefficients (absolute value) in the PCA-based model for the prediction of the neuropsychological scores in the verbal memory domain, and model regression coefficients. Black stars represent coefficients = 0.

## Data Availability

The code used to run the analyses is publicly available at https://github.com/fcalesella/ccn_project.

## Abbreviations

RSFC: Resting state functional connectivity
LASSO: Least absolute shrinkage and selection operator
PCA: Principal component analysis
ICA: Independent component analysis
DL: Dictionary learning
NNMF: Non-negative matrix factorization
fMRI: Functional magnetic resonance imaging
MSE: Mean squared error
CV: Cross-validation
LOO: Leave-one-out
n-LOO: Nested leave-one-out
BIC: Bayesian information criterion
EEG: Electroencephalography
