# Supplementary figures and images for: A comparison of feature extraction methods for prediction of neuropsychological scores from functional connectivity data of stroke patients
Source: Brain Inform. 2021 Apr 20;8(1):8. doi: 10.1186/s40708-021-00129-1 (PMC8058135; doi:10.1186/s40708-021-00129-1)

**Feature 2**  
**Weight = -0.4095**

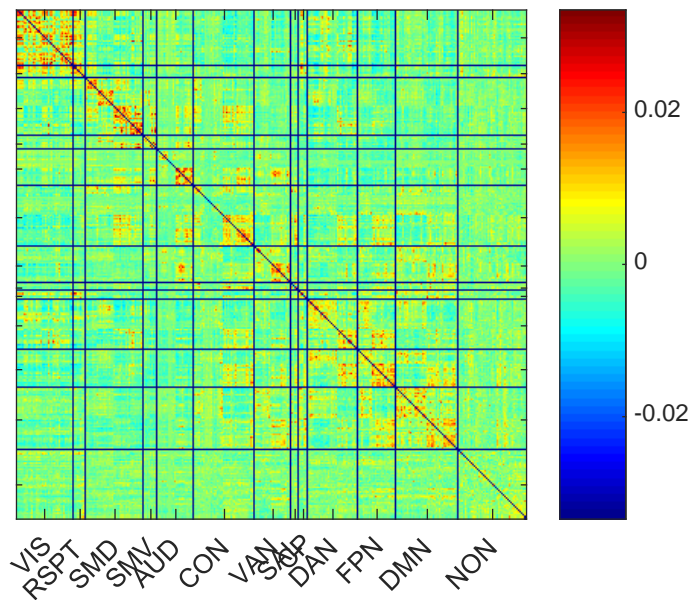

**Feature 9**  
**Weight = 0.3911**

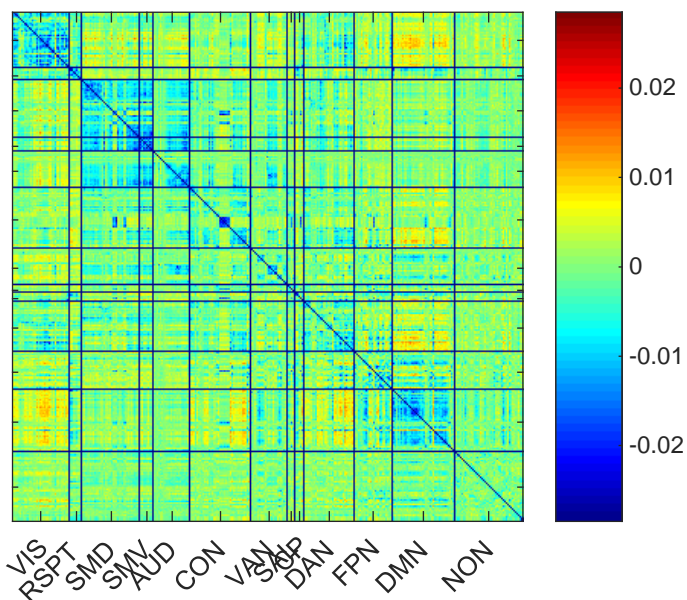

**Feature 27**  
**Weight = -0.2808**

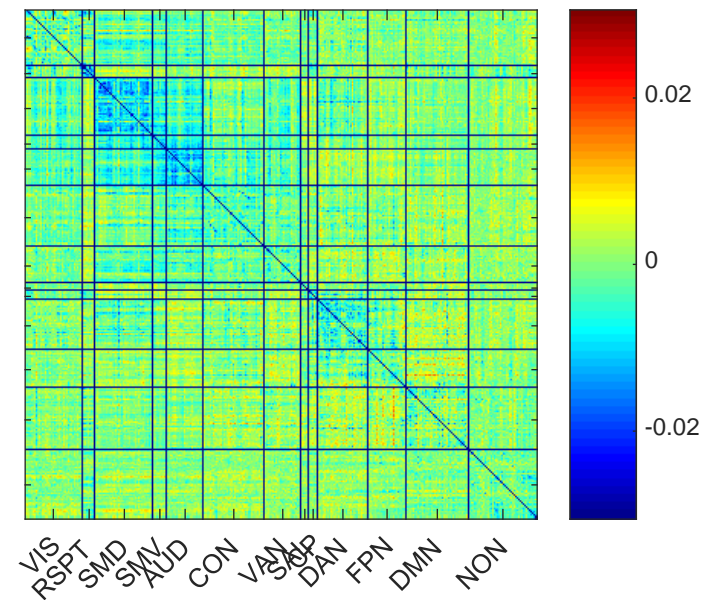

**Feature 28**  
**Weight = -0.1311**

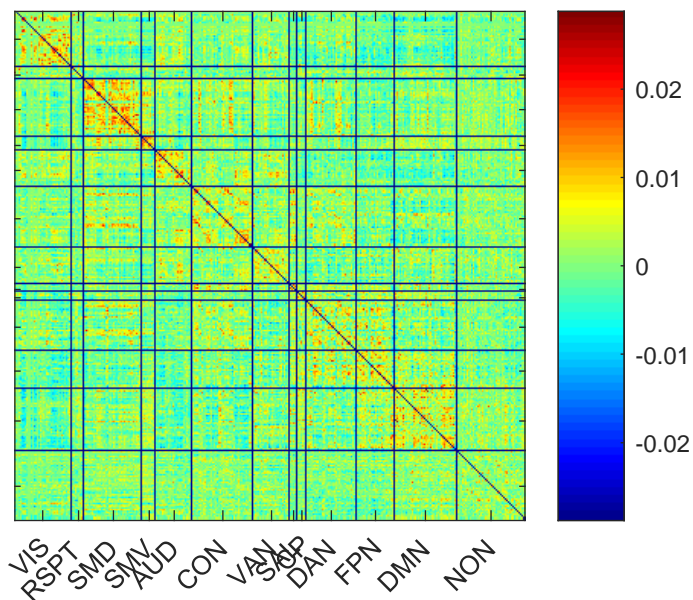

**Feature 26**  
**Weight = 0.1035**

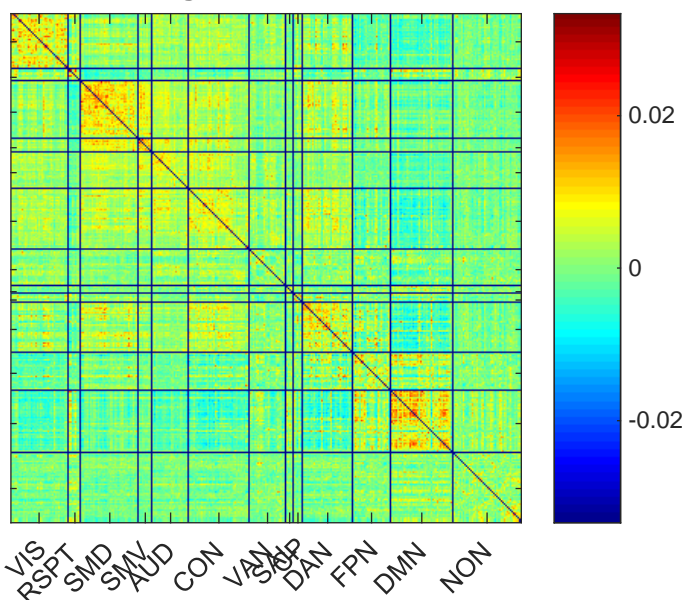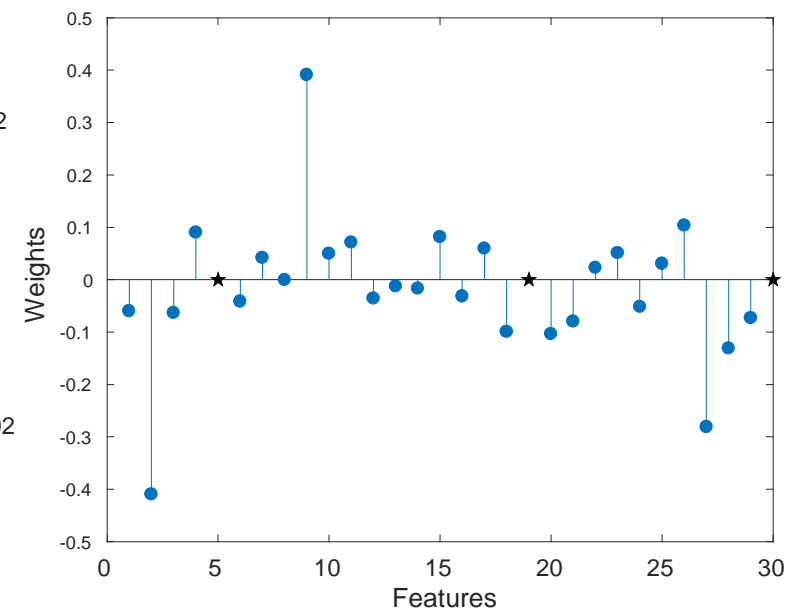

Supplement: Supplementary file 1 — Additional file 1: Figure S1. The 5 features associated to the highest regression coefficients (absolute value) in the DL-based model for the prediction of the language scores, and model regression coefficients. Black stars represent coefficients = 0. [file 40708_2021_129_MOESM1_ESM.pdf]

Feature 16

Weight = -0.3326

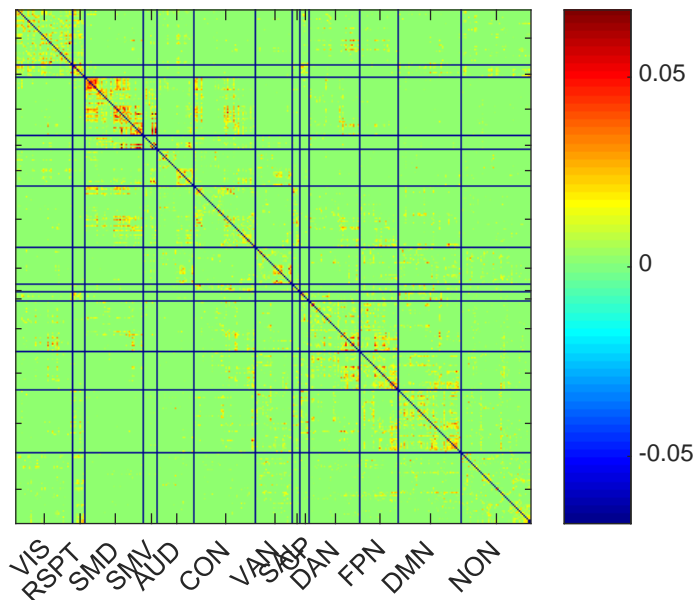

Feature 10

Weight = -0.2924

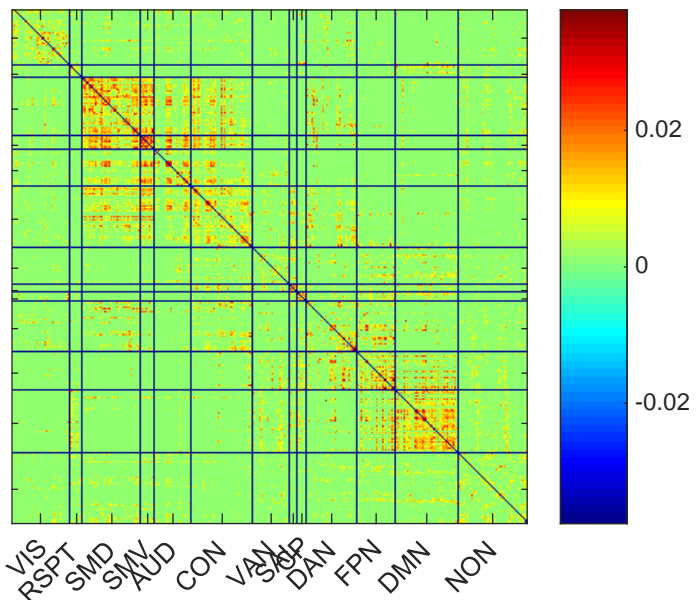

Feature 33

Weight = 0.2012

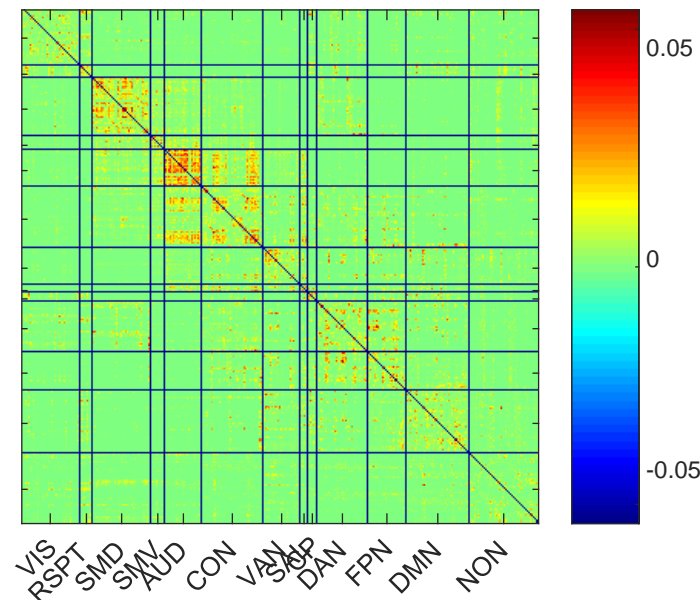

Feature 2

Weight = -0.1880

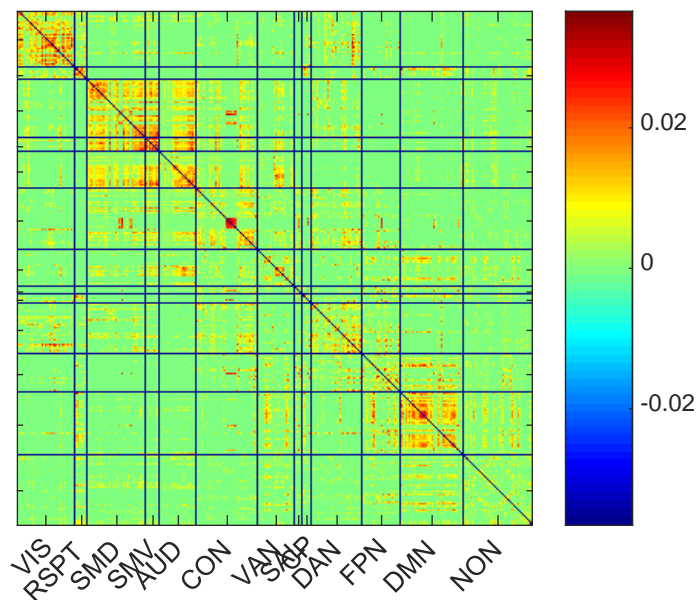

Feature 27

Weight = 0.1564

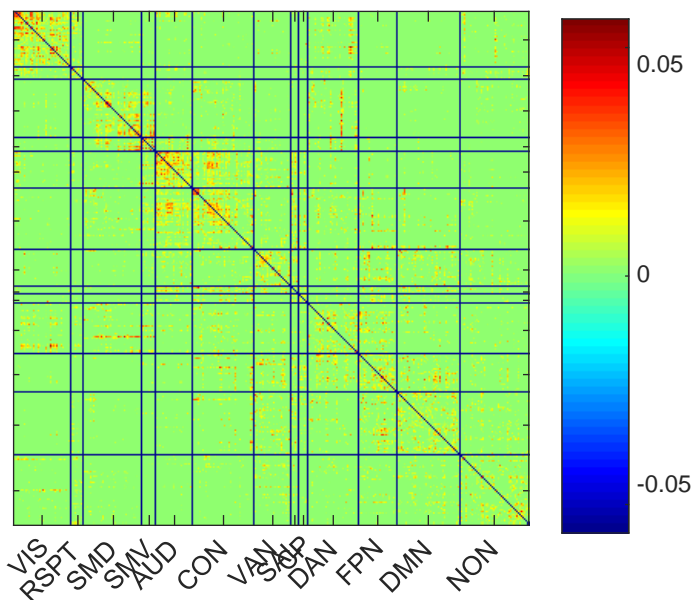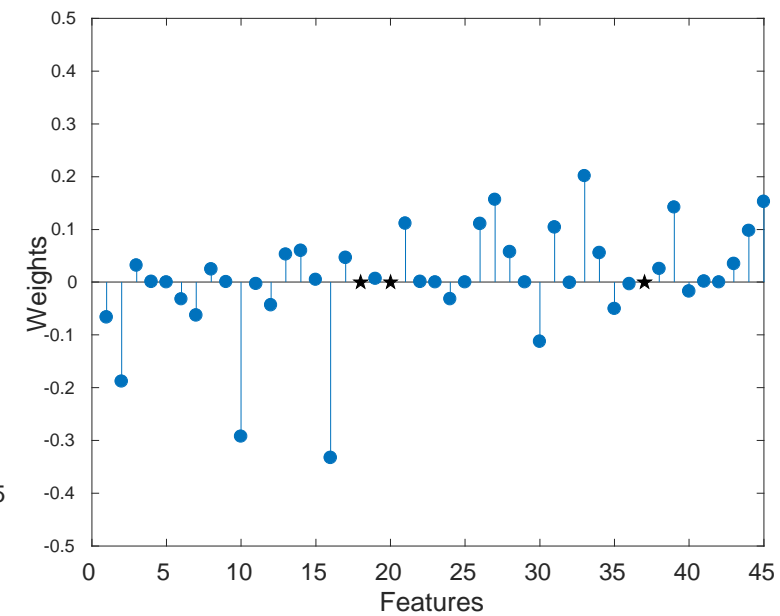

Supplement: Supplementary file 2 — Additional file 2: Figure S2. The 5 features associated to the highest regression coefficients (absolute value) in the NNMF-based model for the prediction of the language scores, and model regression coefficients. Black stars represent coefficients = 0. [file 40708_2021_129_MOESM2_ESM.pdf]

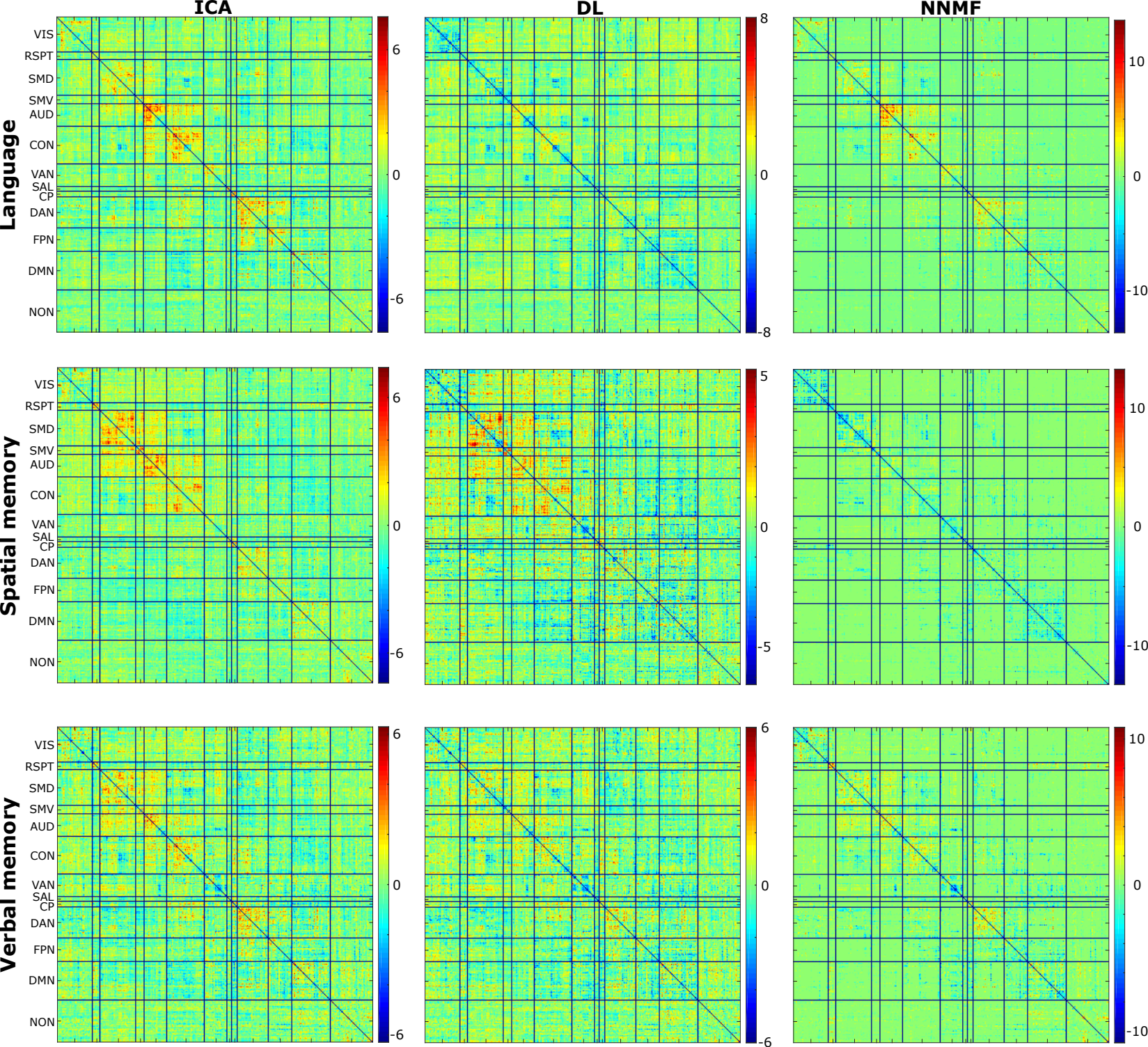

Supplement: Supplementary file 3 — Additional file 3: Figure S3. Maps of predictive functional connectivity edges for ICA-, DL- and NNMF-based models obtained by back-projecting the regression coefficients. DL: Dictionary Learning; ICA: Independent Component Analysis; NNMF: Non-Negative Matrix Factorization. [file 40708_2021_129_MOESM3_ESM.pdf]

Feature 35

Weight = 0.1884

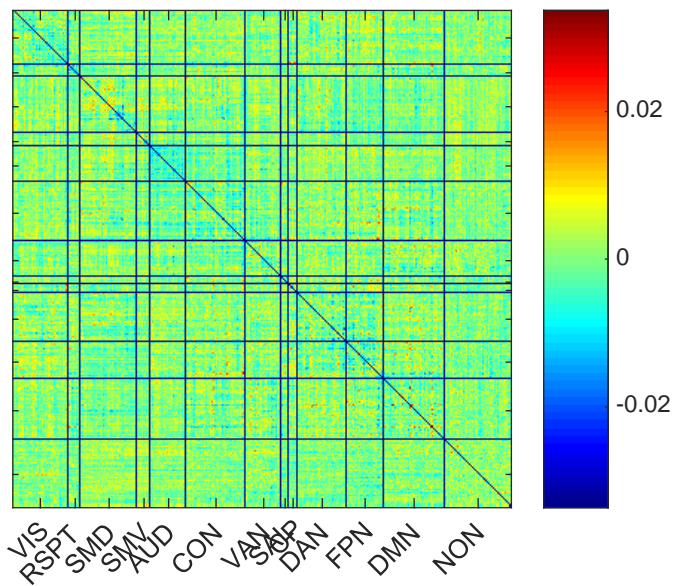

Feature 5

Weight = -0.1880

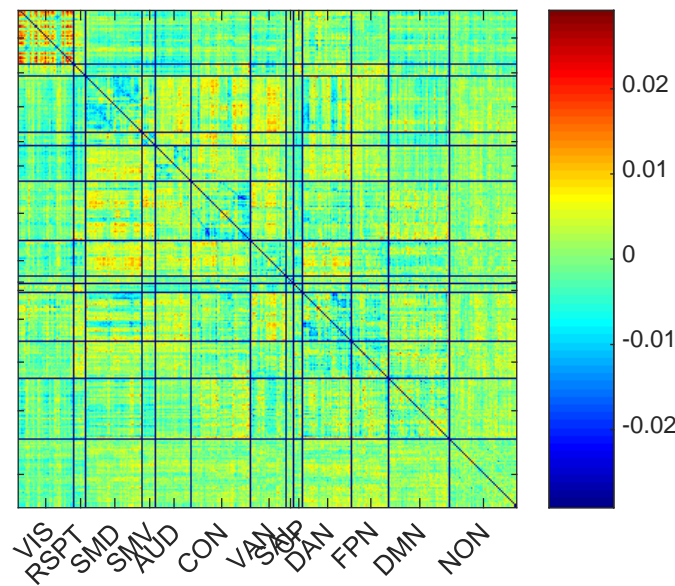

Feature 9

Weight = 0.1792

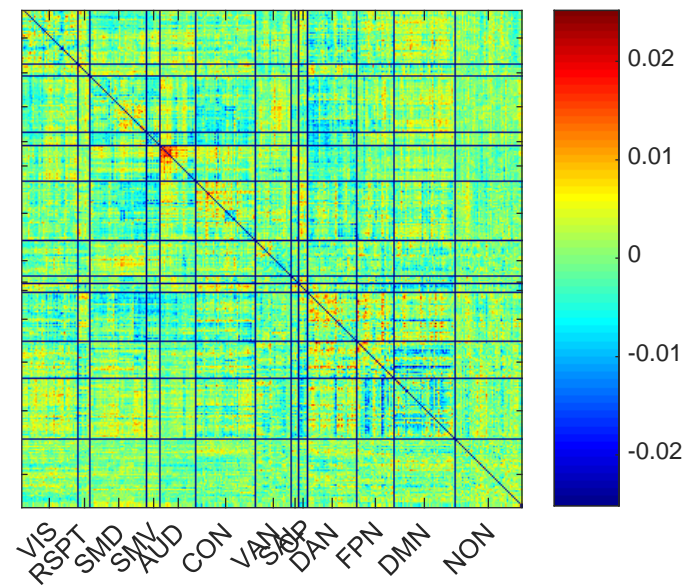

Feature 40

Weight = 0.1310

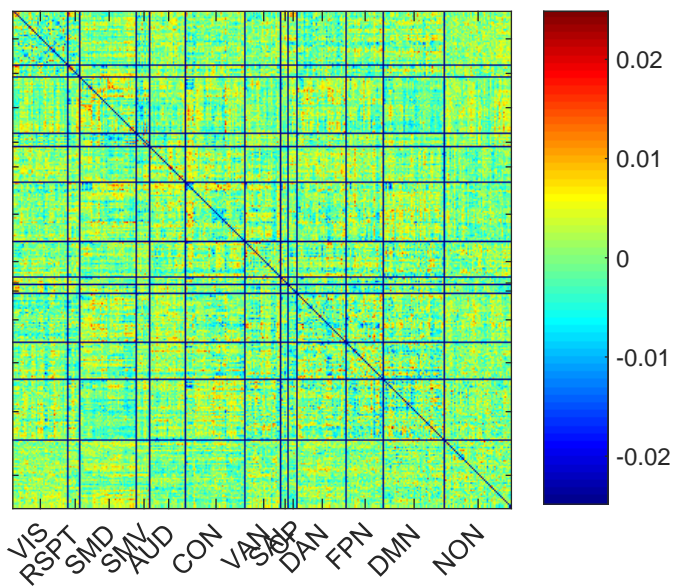

Feature 1

Weight = 0.1216

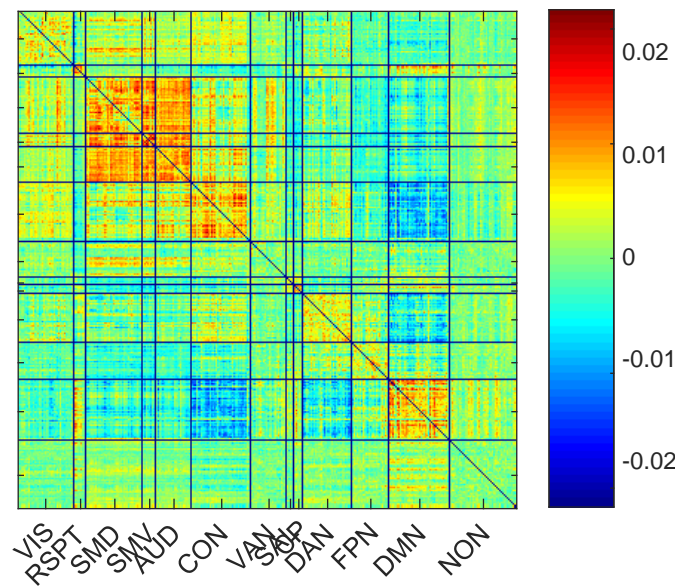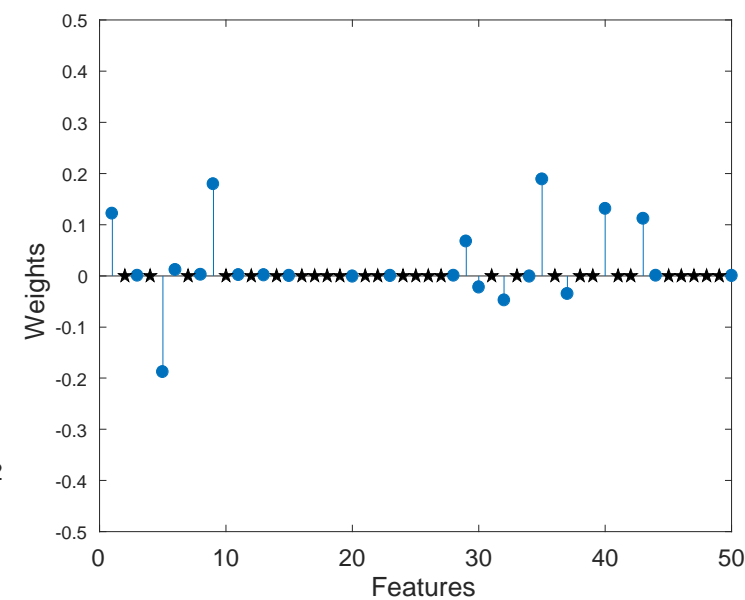

Supplement: Supplementary file 4 — Additional file 4: Figure S4. The 5 features associated to the highest regression coefficients (absolute value) in the PCA-based model for the prediction of the neuropsychological scores in the spatial memory domain, and model regression coefficients. Black stars represent coefficients = 0. [file 40708_2021_129_MOESM4_ESM.pdf]

**Feature 9**  
**Weight = 0.3543**

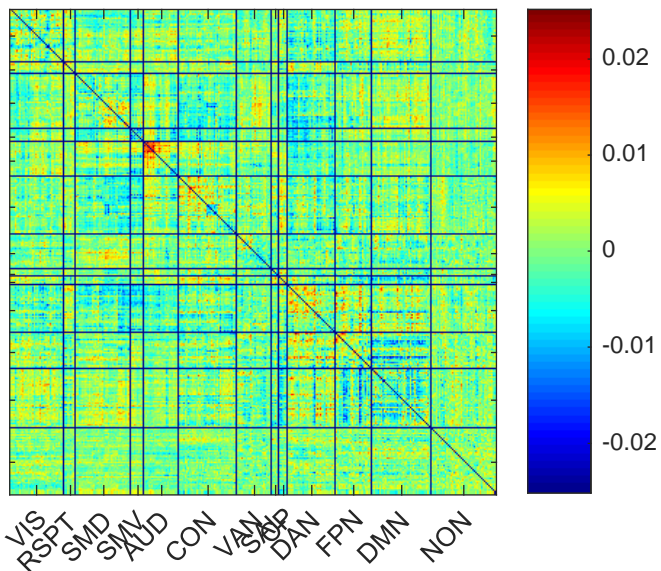

**Feature 35**  
**Weight = 0.2660**

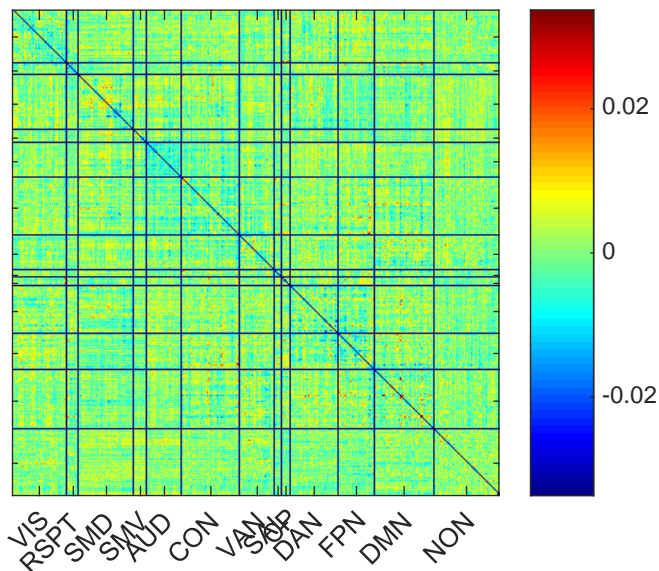

**Feature 20**  
**Weight = -0.1884**

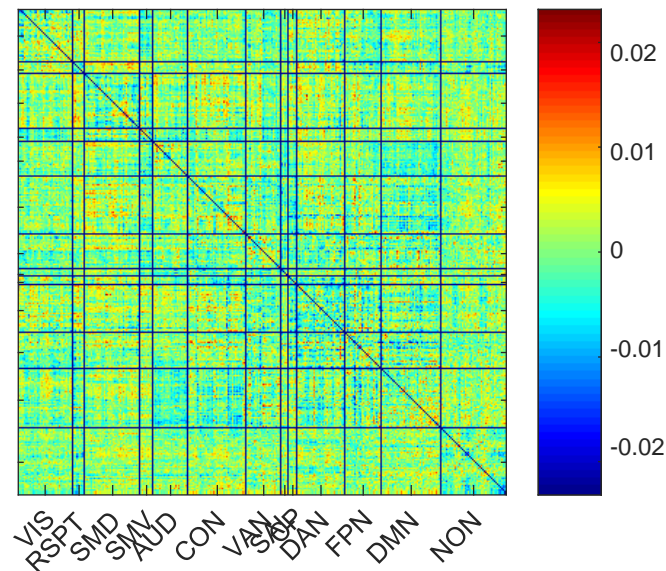

**Feature 40**  
**Weight = 0.1803**

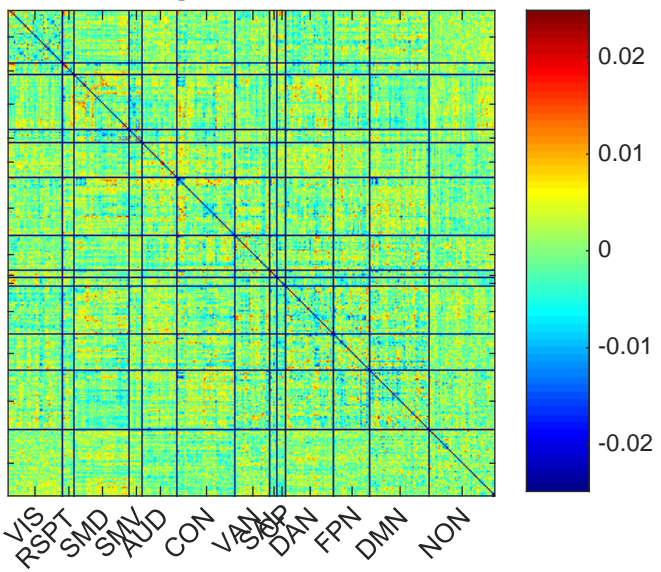

**Feature 41**  
**Weight = -0.1521**

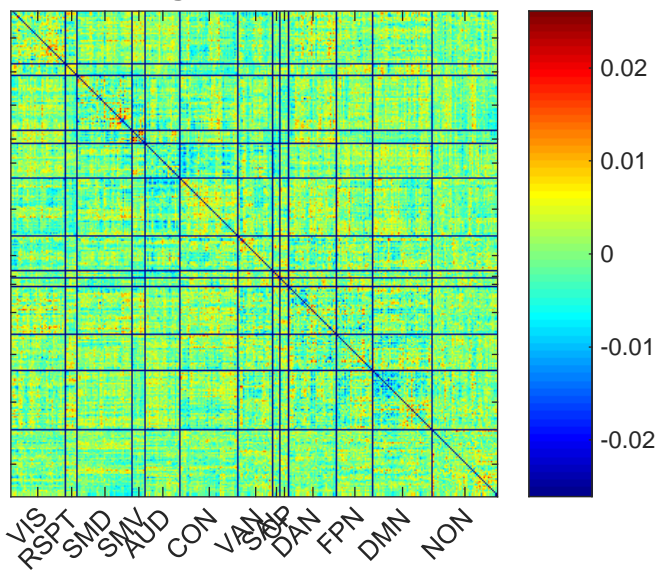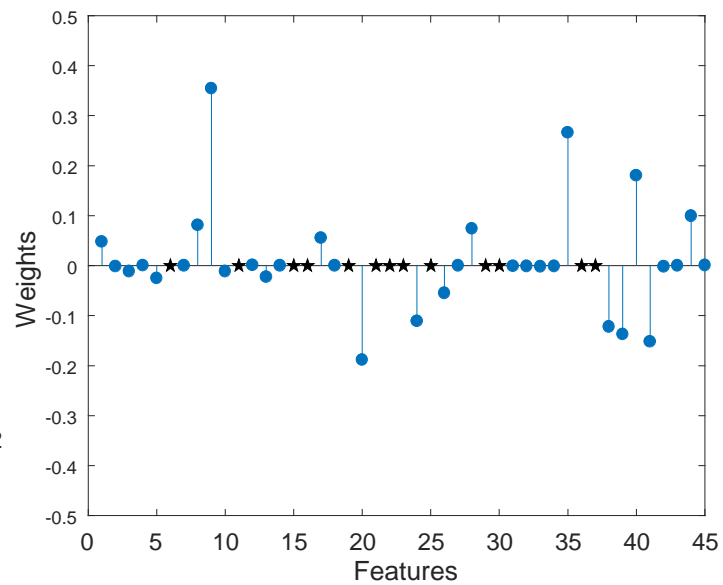

Supplement: Supplementary file 5 — Additional file 5: Figure S5. The 5 features associated to the highest regression coefficients (absolute value) in the PCA-based model for the prediction of the neuropsychological scores in the verbal memory domain, and model regression coefficients. Black stars represent coefficients = 0. [file 40708_2021_129_MOESM5_ESM.pdf]
